# SNPLims: a data management system for genome wide association studies

**DOI:** 10.1186/1471-2105-9-S2-S13

**Published:** 2008-03-26

**Authors:** Alessandro Orro, Guia Guffanti, Erika Salvi, Fabio Macciardi, Luciano Milanesi

**Affiliations:** 1 Consorzio Interuniversitario Lombardo per l'Elaborazione Automatica, Via Sanzio Raffaello 4, 20090 Segrate (MI), Italy; 2 Dipartimento di Scienze e Tecnologie Biomediche, Università degli Studi di Milano, Via Fratelli Cervi 93, 20090 Segrate (MI), Italy; 3 Istituto di Tecnologie Biomediche, Consiglio Nazionale delle Ricerche, Via Fratelli Cervi 93, 20090 Segrate (MI), Italy

## Abstract

**Background:**

Recent progresses in genotyping technologies allow the generation high-density genetic maps using hundreds of thousands of genetic markers for each DNA sample. The availability of this large amount of genotypic data facilitates the whole genome search for genetic basis of diseases.

We need a suitable information management system to efficiently manage the data flow produced by whole genome genotyping and to make it available for further analyses.

**Results:**

We have developed an information system mainly devoted to the storage and management of SNP genotype data produced by the Illumina platform from the raw outputs of genotyping into a relational database.

The relational database can be accessed in order to import any existing data and export user-defined formats compatible with many different genetic analysis programs.

After calculating family-based or case-control association study data, the results can be imported in SNPLims. One of the main features is to allow the user to rapidly identify and annotate statistically relevant polymorphisms from the large volume of data analyzed. Results can be easily visualized either graphically or creating ASCII comma separated format output files, which can be used as input to further analyses.

**Conclusions:**

The proposed infrastructure allows to manage a relatively large amount of genotypes for each sample and an arbitrary number of samples and phenotypes. Moreover, it enables the users to control the quality of the data and to perform the most common screening analyses and identify genes that become “candidate” for the disease under consideration.

## Background

Genome wide search for genes underlying common diseases is enormously facilitated by the use of high throughput genotyping. Nowadays, huge amount of molecular markers are available for the human genome and laboratories equipped with recent genotyping technologies can use them to quickly generate hundreds of thousands of genotypes for each DNA under study.

In particular, Single Nucleotide Polymorphisms (SNPs) are one of the most common forms of human genetic variation that can be used to discover the sequence variants affecting common diseases by examining them for statistically significant association with measurable phenotypes.

In a typical molecular biology laboratory genotype data are usually managed with the help of specialized software (LIMS - Laboratory Information Management Systems) that implements several useful functions, for example: sample tracking for all steps of the experiments, clustering of fluorescent values, visualization and manual correction of genotypes with ambiguous assignment, generation of genotype reports.

Some genotype management systems have been implemented in last years with different features and supporting different genotyping technologies (GenoDB [1], PacLIMS [2], SNPP [3], TIMS [4], [5], [6]). Even though they are useful tools, unfortunately, none of these available systems seem to be easy to customize or integrate in pre-existent infrastructures. Since the software provided together with our microarray platform (Illumina [7]) is suitable for managing raw genotype data, we started to develop a system mainly devoted to the management of post-genotyping activities with particular emphasis to the support of the most common analysis performed in association studies.

In particular the integration in a unique database of genotype, phenotype and demographic data coming from different laboratories facilitates the generation of reports for both visualization and data input for further analysis.

The main features of the system are: automatic import of genotype data from the Illumina microarray platform; definition and assignment of phenotypes to the subjects, including both qualitative and quantitative traits; control of the quality of the data in order to select markers with high genotyping score; statistical descriptive analysis that provides information about basic features and quality of data; analysis of the genetic population structure to identify stratification; statistical descriptive analysis that provides information about basic features and quality of data; single point analysis of association between genotype and quantitative or qualitative traits; multi locus analysis to combine genotypes of adjacent markers and find associations between haplotypes and phenotypes.

## Implementation

The system has been implemented as a client/server application and deployed in a Debian Linux server [8] in which the main storage element is a PostgreSQL database [9] accessed through a web application written with the Zope Web Application Framework [10]. Users can access to the data in two ways: through a command line client within the Linux server and through a web interface. The first method is useful when other command line applications or scripts need to be integrated in pipelines for automatic computation; the second approach is more user oriented and it is used especially for visualization and data management.

Access policy is managed with a mixed approach based on system user accounts and Zope object permissions. Objects stored in the database are grouped in logical sessions that represent data acquisitions or computation results so that multiple studies can be managed in logical projects and shared between users. For example a genotyping session can represent the acquisition into the database of a group of DNA genotypes related to the same study project.

### System architecture and data model

**Figure 1:**
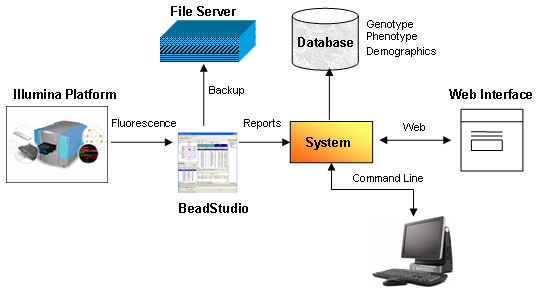
**System Architecture**. General schema of the management of raw data from the Illumina Platfom to the database.

Although the system is mainly devoted to the management of SNP data produced with the Illumina platform [7], this is not a strict requirement. Other types of SNP genotyping technologies can be added in the system using suitable XML descriptors. The main data flow of the system is shown in Figure 1. The raw data represented by image files of the fluorescence values are managed directly by the software distributed together with the machine (BeadStudio software package) and stored for backup. Files containing numerical fluorescence values and genotypes, one for each DNA sample and for each marker, are parsed and inserted in the database together with the information related to the genotyping quality (gcscore – GenCall score). Genotype data are expressed both in general biallelic format (AA, AB, BB, 00=missing) and base biallelic format (for example GG, GT, TT, 00=missing). In a typical genome wide study, for example using the HumapHap300 for 300 subjects, about 90 million of records will be generated in the genotyping session.

Similarly it is possible to define simple phenotype attributes related to individuals and to store them in the database. Phenotypes can be related both to the disease status of subjects (case/control studies) and to a numeric quantitative trait. A phenotype is defined through a unique name, a data type and the data structure (table structure) in which it will be stored. The most common data types (numerical, categorical and strings) supported by the database management system are also supported by the infrastructure. Each phenotype value is stored together with the phenotype ID, the individual ID and the session ID which represent a logical group of values (usually referred to the same population). In this way it is possible to define multiple phenotypes associate them to individuals.

Demographic attributes are related to the parental relationship between the subjects and to the race of the subjects. They are managed like the phenotype attributes but it is not possible to define acquisition session in this case because they are strictly related to the subject and not estimated.

**Figure 2:**
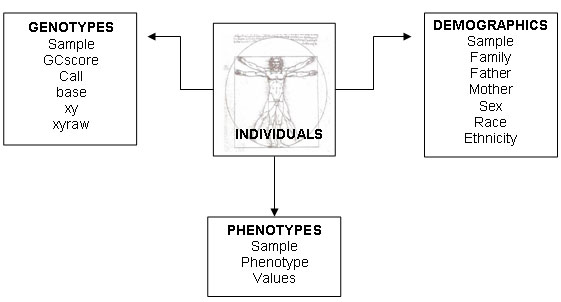
**Data model for genotype, phenotype and demographic data.** Data model of the main database. Individuals are annotated with three types of information: genotypes, phenotypes and demographics.

Figure 2 shows the data model of the system in which the individual, the central object of the model, is characterized with information of the three types described above.

### Analysis

**Figure 3:**
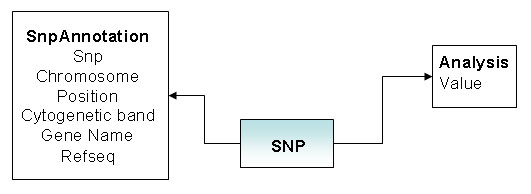
**Data model for the analysis results.** Data model for the analysis database. SNPs are annotated with information about intrinsic features of the marker and the results of analysis.

Analyses supported by the system are mainly focused on genome wide association. In particular for each supported tool the input can be generated automatically from the raw data and the output of analyses imported and indexed in the database. The data model for representing the results of a genome wide analysis is shown in Figure 3. Each SNP can be annotated with two types of values: values representing the result of the analysis (for example the allelic p value of Hardy-Weinberg Equilibrium test or the genetic association) and values representing the intrinsic attributes of the SNP (for example the chromosome, the map position, gene).

In this way it is possible to rank significant results and use relative SNP to generate other inputs for further analyses. The list of supported tools is shown in Table 1.

**Table 1:** List of supported tools

**Tool**	**Ref**	**Description**
Plink	[11]	Whole Genome Association Analysis Toolset
eigenstrat	[13]	Software for detecting and correcting for population stratification in genome-wide association studies
structure	[14]	Software package for using multi-locus genotype data to investigate population structure
Fbat	[15]	Software for implementing family-based association tests
WGAViewer	[16]	Software tool for genomic annotation of whole genome association studies
Haploview	[17]	Tool for analysis and visualization of LD and haplotype maps
phase	[18]	Software for haplotype reconstruction, and recombination rate estimation from population data
PedSplit	[19]	Pedigree Management for stratified analysis

Similarly to the genotype and phenotype acquisition, all analysis results can be grouped in sessions that represent a logical unit of analysis (for example the analysis of group of DNA samples or of a particular cytogenetic region of interest).

### Reports generation

The system can export three types of reports:

– *Input reports* are used to produce file input for analysis tools. They are specific for the particular program and the most common is the ped format that integrates in a unique file pedigree data, genotypes and phenotypes.

– *CSV reports* are useful to import data in a calc-sheet software (like Excel or StataSE) or as general purpose input format for R or Matlab.

– *Graphical reports* are mainly graphical plots of values along a chromosome region (for example the p value of Hardy-Weinberg test or the association test).

Table 2 shows all the export formats supported by the system. In particular, both CSV and input reports can be visualized and saved in local file. More information about the report generation will be shown in the result session.

**Table 2:** List of supported export format

**Format**	**Description**
**Quality control and summary statistics**	
gcscore	List of ‘GenCall scores’ of selected samples
info	Marker information file (*Haploview*)
map	Marker information file (*PLINK*)
ped	Linkage Pedigree format (*Haploview/PLINK*)
Pedallelic/pedgenot	Linkage Pedigree format (*StataSE*)
**Family based association**	
fbat	Input for implementing family-based association tests (*fbat*)
**Population Stratification**	
eigenstrat	Input files of genotypes and phenotypes (*EIGENSTRAT*)
**Haplotyping**	
phase	Input for reconstructing haplotype (*phase*)
**Annotation**	
wgaviewer	Input for genomic annotation (*WGAViewer*)
**Others**	
xyraw	Report for Pooling Statistics (*R*, *StataSE*)

### Web Interface and Client

The web interface has been implemented with the Zope Framework and in particular using the Plone content management product [12]. In this way some functionality like the management of users, permissions and document workflows are inherited directly from the underlying framework.

**Figure 4:**
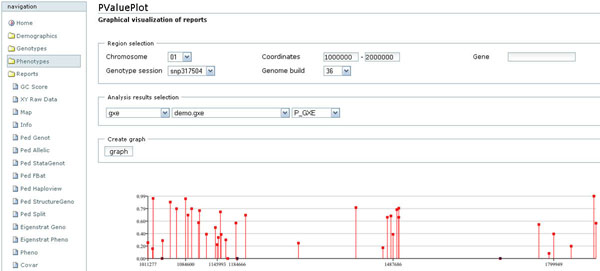
**Web Interface**. A screen shot of the web interface showing the graphical representation of the p value of a statistical analysis along the chromosome.

The web interface is composed by four main tab containing: demographics, genotypes, phenotypes, reports. The first three contain interfaces for managing the respective data types, defining acquisition sessions, and visualizing summary statistics (see result section for examples). The reports tab contains web page for generate reports as described in the previous paragraph. All reports can be both visualized in the browser and exported as file in the server. In addition users can access to the same functionalities through a command line application installed in the server. Figure 4 shows a screen shot of the web interface.

### Performances

The software is installed on Intel(R) Xeon(TM) CPU 2.40GHz (1G RAM) on the Debian (kernel 2.6) operating system. In the current installation the creation of a report integrating results of analysis with SNP annotation takes a time negligible respect to the creation of a PED input which takes about 10 min for a file 100 samples and 300k SNPs. The association case/control analysis performed on the same dataset with plink takes about 2 min.

## Results

In this session we describe the context in which the proposed system has been developed and tested. Genotype data, produced with the HumanHap300 (317k SNPs), for 95 case subjects and 91 controls has been used for a genome wide association study search in order to find regions or genes related to the schizophrenia disease.

The system has been used for both managing data and supporting statistical analysis. In particular descriptive statistics has been used to summarize and describe the main statistical properties of data whereas inferential statistics, concerning the inference of new insights about the genetic association, has been used for the screening. The analysis pipeline includes the quality control and the summary statistics of raw data as descriptive statistics and analysis of population stratification and association test between genotype and phenotype as inferential statistics. Reports of computed statistical parameters are integrated with the SNP annotation of the HumapHap300 in order to compare regions with high significance with the biological properties of the regions.

### Descriptive statistics

Descriptive statistics are used to describe the basic features of the data and to perform the quality control of raw data produced by the genotyping platform.

The system supports the evaluation of the *call rate* parameter that counts the number of called SNPs per sample and the *GenCall score* calculated by the BeadStudio software that indicates the quality of the SNP clustering. They are useful measures to evaluate the global quality of the genotyping.

**Figure 5:**
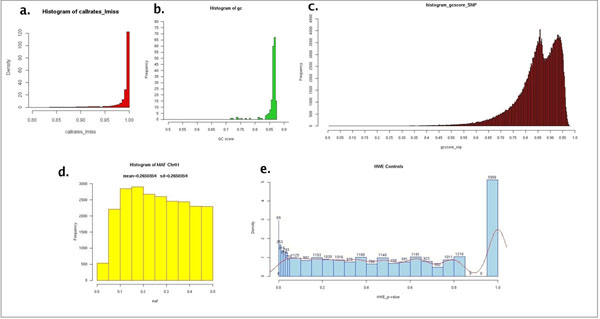
**Summary statistics**. Examples of histograms of summary statistics for quality control: a) histogram of Call Rates; b) Histogram of GenCall Scores per sample; c) Histogram of GenCall Scores per SNPs; d) Histogram of Frequency of Minor Allele (MAF); e) Histogram of Hardy Weinberg P values (HWE) of control individuals.

Moreover the system helps to manage the output of standard summary statistics (generated by statistical tools) as the “missing genotype rate” (proportion of missing SNPs or missing samples), the “minor allele frequency” (ratio of less common allele variant to the more common allele variant) and the “Hardy-Weinberg equilibrium” test (calculation of chi-square test for deviation from HWE). Summary statistics are useful for checking the genotypes in terms of the expected quality on the following analysis results. In Figure 5 are shown some global quality reports in a typical genotyping session.

### Inferential statistics

**Figure 6:**
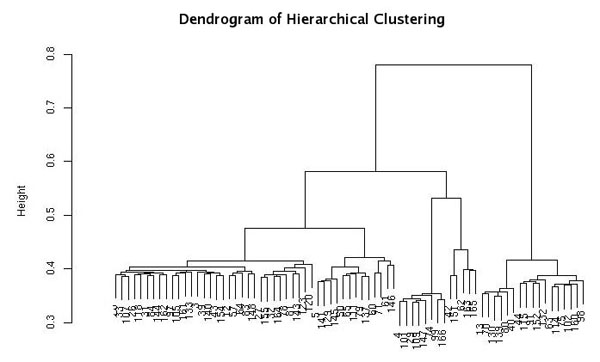
**Population Stratification**. Example of clustering dendrogram of inferred population structure. The complete linkage agglomerative clustering, based on pairwise identity-by-state (IBS), was obtained with PLINK.

The system supports the management of input-output files of population stratification analysis. Population stratification can occur in case-control association studies when allele frequencies differ between cases and controls because of systematic differences in ancestry. It may lead to false positive associations due to population structure rather than association of genes with the disease. In order to infer the structure of population we apply many tools as Plink, EIGENSTRAT, Structure, Fst, Genomic Control. In Figure 6 the clustering dendrogram of inferred population structure is shown.

In order to identify a set of markers with high degree of statistical significance for the disease, the following association tests has been performed: the basic association test for a disease trait based on comparing allele frequencies between cases and controls, the Cochran-Armitage trend test, different genetic models (dominant, recessive and general), tests for stratified samples and a test for a quantitative phenotype.

### Association and annotation

**Figure 7:**
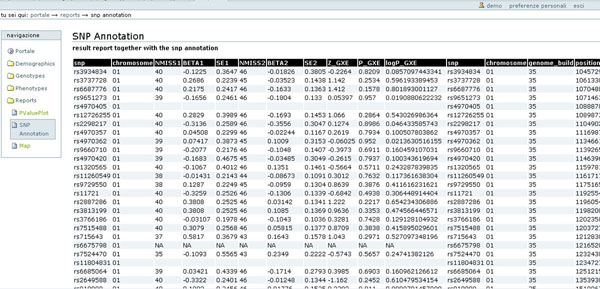
**SNP Annotation.** Tabular representation of the analysis annotated with the SNP information.

Integration of association results and the SNP information of the HumapHap300 can be obtained in a tabular form. This report allows visualizing information about every SNP (chromosome, position, cytogenetic band, gene name, etc) together with the results of multiple analysis and in order to selecting regions of interest. Figure 7 shows the table generated for a region of chromosome 1.

## Discussion and conclusions

In this paper a system for data management of genotypes and phenotype data has been proposed. Main focus of the infrastructure is the support of genetic studies of genome-wide association studies by wrapping the most common tools used in this field.

## Availability and requirements

Project name: SNPLims

Project homepage: 

Operating system(s): tested for Debian.

Programming language: Python 2.4, Zope 2.9 and Plone 2.5

Database management system: PostgreSQL 8.1

## Competing interests

The authors declare that they have no competing interests

## Authors' contributions

AO designed the database management system, wrote the source code of the infrastructure and wrote the first draft of the manuscript. FM coordinated the analysis. LM coordinated the design and implementation of the system. GG and ES performed the analysis described in the results session. All authors participated in the drafting of the manuscript and approved the final version.
